# Assessment of the prognostic impact of the Epstein–Barr virus-encoded latent membrane protein-1 expression in Hodgkin's disease

**DOI:** 10.1054/bjoc.2001.1774

**Published:** 2001-05

**Authors:** M Glavina-Durdov, J Jakic-Razumovic, V Capkun, P Murray

**Affiliations:** Department of Pathology andDepartment of Nuclear Medicine University Hospital Split, Institute of Pathology Clinical Medical Center ‘Rebro’ Zagreb, Croatia, Department of Pathology, Division of Cancer Studies, University of Birmingham, Birmingham, B15 2TT, UK

## Abstract

We have examined expression of the Epstein–Barr virus (EBV) latent membrane protein-1 (LMP1) in the malignant Hodgkin and Reed–Sternberg (HRS) cells of Hodgkin's disease (HD) and its impact on response to treatment and on survival. Paraffin tissue from 100 adult immunocompetent patients with HD were analysed using immunohistochemistry to identify LMP1 expression. According to the Rye classification, 8% of patients had lymphocyte predominance (LP) subtype, 48% had nodular sclerosis (NS) disease, 37% were of the mixed cellularity (MC) subtype and 7% were of the lymphocyte depletion (LD) subtype. During the five year follow-up period 27 patients died and 74 patients achieved a complete remission. Patients with LD subtype were older (*P* = 0.03), less frequently achieved complete remission (*P* = 0.01), had shorter disease-free survival (*P* = 0.01) and overall survival (*P* = 0.002) compared with the other subtypes of HD. LMP1 expression was found in the tumour cells of 26% of cases of HD. LMP1 expression was less common in NS disease than in the other subtypes (*P* = 0.05), whereas an association between MC subtype and LMP1 expression was not found (*P* = 0.22). Using the log-rank test there were no differences in overall survival or disease-free survival based on EBV status either when all patients were analysed or when LD and LP subtypes were excluded. However, the presence of EBV was associated with significantly longer disease-free survival in the subgroup of patients ≤ 30 years old (*P* = 0.02) and in those patients ≤ 34 years old (*P* = 0.05). In contrast, there was a trend for shorter disease-free survival for EBV-positive patients in the subgroup > 35 years old, but this difference was not statistically significant (*P* = 0.11). A similar trend was observed in patients > 50 years old. Analysis of the impact of LMP1 expression in patients who had different stage and B symptoms status showed that expression of EBV was associated with longer disease-free survival (*P* = 0.019) in early stage (1 + 2) patients without B symptoms. Significant differences in the other subgroups based on EBV status was not found. Factors adversely affecting the likelihood to achieve a complete remission were: absence of LMP1 expression (OR 6.4, 95% Cl 1.07–38.5, *P* = 0.04), age (OR 1.68, 95%Cl 1.15–2.5, *P* = 0.007) and subtype of HD (OR 3.32, 95%Cl 1.11–9.94, *P* = 0.03). Age and subtype of HD had an independent impact on overall survival (*P* = 0.01). We conclude that expression of LMP1 in HRS cells has a favourable impact on prognosis for HD patients, but that this effect may be restricted to young adult patients and those with early stage disease. © 2001 Cancer Research Campaign http://www.bjcancer.com


*British Journal of Cancer* (2001) **84**(9), 1227–1234
© 2001 Cancer Research Campaign
doi: 10.1054/ bjoc.2001.1774, available online at http://www.idealibrary.com on IDEAL®

http://www.bjcancer.com

### Assessment of the prognostic impact of the Epstein–Barr virus-encoded latent membrane protein-1 expression in Hodgkin's disease

**M Glavina-Durdov[1], J Jakic-Razumovic[3], V Capkun[2] and P Murray[4]**

[1]Department of Pathology and [2]Department of Nuclear Medicine University Hospital Split, [3]Institute of Pathology Clinical Medical Center 'Rebro' Zagreb, Croatia, [4]Department of Pathology, Division of Cancer Studies, University of Birmingham, Birmingham, B15 2TT, UK

**Summary** We have examined expression of the Epstein–Barr virus (EBV) latent membrane protein-1 (LMP1) in the malignant Hodgkin and Reed–Sternberg (HRS) cells of Hodgkin's disease (HD) and its impact on response to treatment and on survival. Paraffin tissue from 100 adult immunocompetent patients with HD were analysed using immunohistochemistry to identify LMP1 expression. According to the Rye classification, 8% of patients had lymphocyte predominance (LP) subtype, 48% had nodular sclerosis (NS) disease, 37% were of the mixed cellularity (MC) subtype and 7% were of the lymphocyte depletion (LD) subtype. During the five year follow-up period 27 patients died and 74 patients achieved a complete remission. Patients with LD subtype were older ($P$ = 0.03), less frequently achieved complete remission ($P$ = 0.01), had shorter disease-free survival ($P$ = 0.01) and overall survival ($P$ = 0.002) compared with the other subtypes of HD. LMP1 expression was found in the tumour cells of 26% of cases of HD. LMP1 expression was less common in NS disease than in the other subtypes ($P$ = 0.05), whereas an association between MC subtype and LMP1 expression was not found ($P$ = 0.22). Using the log-rank test there were no differences in overall survival or disease-free survival based on EBV status either when all patients were analysed or when LD and LP subtypes were excluded. However, the presence of EBV was associated with significantly longer disease-free survival in the subgroup of patients ≤ 30 years old ($P$ = 0.02) and in those patients ≤ 34 years old ($P$ = 0.05). In contrast, there was a trend for shorter disease-free survival for EBV-positive patients in the subgroup > 35 years old, but this difference was not statistically significant ($P$ = 0.11). A similar trend was observed in patients > 50 years old. Analysis of the impact of LMP1 expression in patients who had different stage and B symptoms status showed that expression of EBV was associated with longer disease-free survival ($P$ = 0.019) in early stage (1 + 2) patients without B symptoms. Significant differences in the other subgroups based on EBV status was not found. Factors adversely affecting the likelihood to achieve a complete remission were: absence of LMP1 expression (OR 6.4, 95% Cl 1.07–38.5, $P$ = 0.04), age (OR 1.68, 95%Cl 1.15–2.5, $P$ = 0.007) and subtype of HD (OR 3.32, 95%Cl 1.11–9.94, $P$ = 0.03). Age and subtype of HD had an independent impact on overall survival ($P$ = 0.01). We conclude that expression of LMP1 in HRS cells has a favourable impact on prognosis for HD patients, but that this effect may be restricted to young adult patients and those with early stage disease. © 2001 Cancer Research Compaign. http://www.bjcancer.com

Hodgkin's disease (HD) is a lymphoid neoplasm characterized by a low frequency of malignant Hodgkin and Reed–Stemberg (HRS) cells in an abundant background of reactive inflammatory cells. Active accumulation of these reactive cells, which include lymphocytes, eosinophils, monocytes, neutrophils and fibroblasts, is dependent upon antigen presentation and activation of HRS cells and bystander reactive cells (Gruss et al, 1997). In the majority of cases HRS cells are believed to derive from germinal centre or post-germinal centre B cells that carry non-functional $V_H$ gene rearrangements, suggesting that HRS cells can bypass the apoptosis that would otherwise eliminate B cells with defectively rearranged Ig genes (Hummel et al, 1995; Kanzler et al, 1996).

In a variable percentage of cases, Epstein–Barr virus (EBV) latent infection is detectable in the malignant HRS cells of HD. Involvement of EBV in the pathogenesis of HD had been suggested indirectly by serological and epidemiological studies (Levine et al, 1971; Gutensohn and Cole, 1980; Mueller et al, 1989). Subsequently, direct evidence of EBV in tumour tissue was established by Southern blot (Weiss et al, 1987) and polymerase chain reaction (PCR) analysis (Shibata et al, 1991), although neither method was able to localize EBV to specific cell types. Later, the development of in situ hybridization for the detection of the highly abundant EBV early RNAs (EBERs) (Glickman et al, 1988; Wu et al, 1990) and immunohistochemistry for the EBV-encoded latent membrane protein-1 (LMP1) (Pallesen et al, 1991; Murray et al, 1992) enabled the localization of EBV to the malignant HRS cells. Although EBV can also be detected in rare non-malignant 'bystander' lymphocytes within HD tissues, it is the detection of EBV within HRS cells that is necessary to establish a HD case as 'EBV-associated'.

LMP1 has many of the characteristics of a classical oncogene and in particular is transforming when transfected into rodent fibroblasts (Wang et al, 1985). In B-lymphocytes, LMP1 is able to upregulate a number of cellular genes, including the apoptosis-inhibiting *bcl-2* gene (Henderson et al, 1991; Wang et al, 1996) as well as inducing IL-10, a potent inhibitor of T cell activation (Nakagomi et al, 1994). The transforming effects of LMP1 and its particularly high level expression in the HRS cells of EBV-associated HD indicate a role for this protein in the pathogenesis of EBV-positive HD and suggest important biological differences in HD that may be dependent upon EBV status (Oudejans et al, 1997).

*Received 15 June 2000*
*Revised 22 January 2001*
*Accepted 25 January 2001*

*Correspondence to:* M Glavina-Durdov

Initial studies investigating the impact of EBV on clinical outcome did not find any correlation between EBV status and survival in HD patients (Fellbaum et al, 1992; Vestlev et al, 1992; Claviez et al, 1994), although the first of these studies used PCR to detect the virus. Recent larger Spanish and English studies (Morente et al, 1997; Murray et al, 1999) have demonstrated a significant association between detection of EBV in HRS cells and longer overall survival and disease-free interval, respectively. However, two other studies have indicated no effect of EBV status on clinical features or outcome in HD patients (Enblad et al, 1997; Axdorph et al, 1999). In the present study we have examined the influence of EBV LMP1 expression on clinical outcome in a series of HD patients from Croatia. Clinical outcome was determined by 3 measures: complete remission, disease-free survival time and overall survival time, as defined previously (Kaufman and Longo, 1992).

### MATERIALS AND METHODS

Our initial study group comprised 143 consecutive patients diagnosed with HD between 1980 and 1990 in the Institute of Pathology, Clinical Hospital Center Rebro, Zagreb and the Department of Pathology, University Hospital, Split. Clinical data, including treatment and outcome (complete remission, disease-free survival and overall survival in a 5 year follow-up period) were retrieved from the patients' hospital records. After clinicopathologic staging, all patients were treated by the MOPP/ABVD chemotherapy protocol and radiotherapy if necessary. 43 patients were excluded from the study because of incomplete clinical data or insufficient material remaining in the tissue block to allow the EBV status to be determined. The remaining 100 patients were grouped according to the Rye classification as LP, NS, MC or LP subtype. Tumour tissue had been fixed in 10% buffered formalin, dehydrated in gradients of ethanol, cleared in xylene and embedded in paraffin wax.

Before the immunohistochemical assay, 4-micrometre paraffin sections were prepared, deparaffinized, hydrated and washed in Tris-buffered saline (TBS, pH 7.6). Immunohistochemistry for LMP1 was performed using a mouse monoclonal antibody to LMP1 (CS1-4, Dako, Glostrup, Denmark) and labelled streptavidin biotin detection kit, LSAB+ (Dako, Glostup, Denmark). Prior to immunohistochemistry, sections were washed in TBS and pretreated for $3 \times 5$ minutes in a microwave oven at maximum power. Negative controls for immunohistochemistry were consecutive test sections in which primary antibody was replaced with non-immune serum of the same IgG subclass.

For statistical analysis, $\chi^2$ test and Fisher's test were used to compare categorical variables. The Student *t*-test and Mann–Whitney test were used to compare continuous variables. The survival of different groups was analysed by the use of Kaplan–Meier curves and the log-rank test. Overall survival was calculated as the time from diagnosis to death from any cause or to the date of the last known follow up. For patients who responded to therapy, disease-free interval was determined as the time from achieving a complete remission to relapse, death from any cause or the end of follow up. For patients who did not respond to chemotherapy, disease-free interval was recorded as zero. Patients were also grouped according to early versus late stage disease +/– the presence of B symptoms, and the influence of EBV status on disease-free survival in each of these groups was assessed. Multivariate logistic regression analysis was used to identify the factors that were of independent significance in the failure to achieve complete remission. Multivariate Cox's regression analysis was used to identify the factors that impacted on overall survival. The following variables: gender, age in decade intervals from < 20 to < 60 years old, subtype of HD (LP, NS, MC), stage (1, 2, 3, 4), presence of B symptoms (present or absent) and LMP1 expression (positive or negative) were included in these analyses. A difference of $P = 0.05$ or less was considered significant.

### RESULTS

Among the 100 eligible patients, 52% were male and 48% were female. The mean age of all patients was 40 years (range 13–84 years). The age distribution was bimodal with 63% patients under 40 years and 25% older than 50 years. In the 5-year follow up period 27 patients had died and 74 patients achieved a complete remission. According to the Rye classification, 8% of patients were of LP subtype, 48% had NS disease, 37% were of MC subtype and 7% were of LD subtype. Patients with LD disease were older than patients with other subtypes ($P = 0.03$). LD patients also achieved a complete remission less frequently ($P = 0.01$), had a a shorter disease-free survival ($P = 0.01$) and a lower overall survival ($P = 0.002$) than the other subtypes which showed no difference in these variables. The presence of latent EBV infection was detected by immunohistochemistry for LMP1 in 26/100 (26%) cases. Table 1 shows the relationship between the presence of EBV and clinical characteristics such as age, histologic subtype, stage, presence of B symptoms, complete remission, disease-free survival and overall survival. Patients with the NS subtype were more often LMP1-negative ($P = 0.05$), but a significant association between patients with the MC subtype and the presence of EBV was not found ($P = 0.22$).

Outcome analysis according to EBV status was performed on all patients and also when the LD and LP subtypes were excluded. The distinction between non-Hodgkin's lymphoma and LD HD is not clear cut and LP cases were excluded as this disease has been shown to differ morphologically, immunophenotypically and clinically from classic HD (Linden et al, 1988; Harris et al, 1994) (Stoler et al, 1995). Using the log-rank test, we did not find any statistically significant differences in overall survival based on EBV status (Figure 1A and 1B). No significant differences were observed in disease-free survival based on EBV status when all patients were analysed ($P = 0.48$) or when the LP and LD subtypes were excluded ($P = 0.46$) (Figures 2A and 2B). However, when the effect of EBV status was examined within different age groups, longer disease-free survival was observed in LMP1-positive patients ≤ 30 years old ($P = 0.02$) as well as in patients ≤ 34 years old ($P = 0.05$) (Figures 3A and 3B). In contrast, within the group of older patients (> 35 years), those with LMP1 negative disease had longer disease-free interval, although this difference did not reach statistical significance ($P = 0.11$) (Figure 3C). There were insufficient numbers to assess the impact of EBV status in the small subgroup of 10 patients > 50 years old who achieved complete remission. However, longer survival of LMP1-negative patients in this group (36.6 ± 14 months versus 22.6 ± 42 months) was noted.

Table 2 shows the distribution of LMP1 expression according to early (1 + 2) and late (3 + 4) stages of HD and the presence of B symptoms. When disease-free survival was analysed in a subset of 29 patients with early stage (1 + 2) HD without B symptoms it was shown that EBV-positive patients in this subgroup had significantly

 © 2001 Cancer Research Campaign

**Table 1** Differences in variables according to LMP1 expression

| Variable | | Cases | EBV+ | EBV– | Test | *P* |
|---|---|---|---|---|---|---|
| Age (years*) | | 100 | 42.1 ± 17 | 36.06 ± 17 | Mann Whitney | 0.34 |
| B symptoms | | | | | | |
| No | | 52 | 16 | 36 | log rank | 0.12 |
| Yes | | 48 | 8 | 40 | log rank | 0.33 |
| Stage | | | | | | |
| 1 | | | 3 | 14 | | |
| 2 | | | 10 | 15 | | |
| 3 | | | 10 | 33 | $\chi^2$ | 0.11 |
| 4 | | | 3 | 12 | | |
| Stages | | | | | | |
| 1+2 | | 42 | 13 | 29 | log rank | 0.48 |
| 3+4 | | 58 | 13 | 45 | log rank | 0.87 |
| Subtype of HD | | | | | | |
| LP | | 8 | 3 | 5 | | |
| NS | | 48 | 9 | 39 | $\chi^2$ | 0.05 |
| MC | | 37 | 11 | 26 | $\chi^2$ | 0.22 |
| LD | | 7 | 3 | 4 | | |
| Complete remission | | | | | | |
| No | | 26 | 4 | 22 | | |
| Yes | | 74 | 22 | 52 | $\chi^2$ | 0.15 |
| Disease free survival | | (months*) | | | | |
| NS+MC | | 65 | 63.1 ± 36.9 | 51.6 ± 38 | log rank | 0.46 |
| All subtypes | | 74 | 59.5 ± 41.2 | 53 ± 36.9 | log rank | 0.48 |
| | ≤ 30 | 33 | 94 ± 36 | 44 ± 36.5 | log rank | 0.02 |
| Age(years) | ≤ 34 | 37 | 85.4 ± 40 | 48 ± 37.8 | log rank | 0.05 |
| | > 35 | 31 | 45.5 ± 35.7 | 61.45 ± 34.8 | log rank | 0.11 |
| | > 50 | 10 | 22.6 ± 42 | 36.6 ± 14 | | |
| Overall survival | | (months*) | | | | |
| NS+MC | | 65 | 116 | 108 | log rank | 0.30 |
| All subtypes | | 100 | 76 | 77 | log rank | 0.97 |

*Mean.

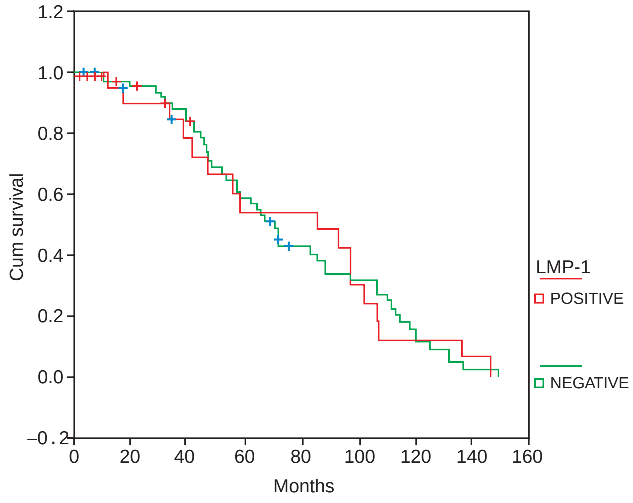

Log rank 0.00 *P* = 0.97

| LMP1 | mean | S.E. | 95% CI |
|---|---|---|---|
| negative | 76 | 5 | 67–86 |
| positive | 77 | 10 | 58–96 |

**Figure 1A** Overall survival in all subtypes of HD according to LMP 1 expression

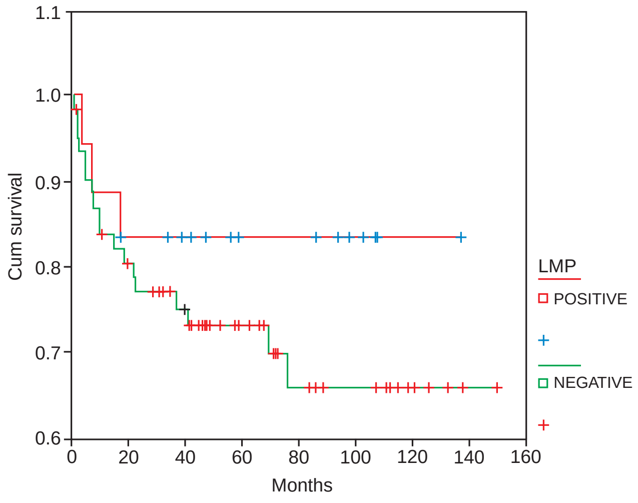

Log rank 1.1 *P* = 0.3

| LMP1 | mean | S.E. | 95% CI |
|---|---|---|---|
| negative | 108 | 8 | 91–124 |
| positive | 116 | 11 | 94–138 |

**Figure 1B** Overall survival in NS and MC subtypes of HD according to LMP 1 expression

*© 2001 Cancer Research Campaign*

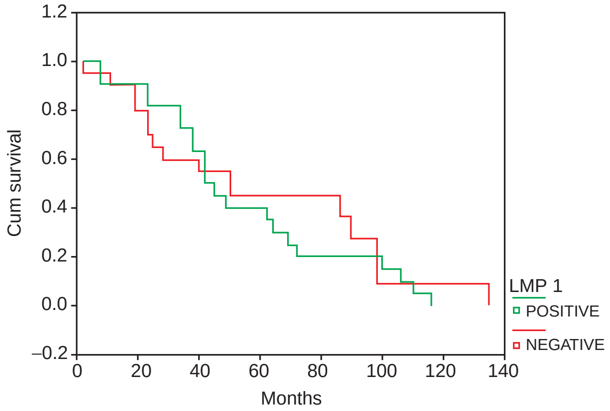

Log rank 0.49 $P = 0.48$

| LMP1 | mean | S.E. | 95% CI |
|---|---|---|---|
| negative | 53 ± 36.9 | 5 | 43–64 |
| positive | 59.5 ± 41.2 | 9 | 41–77 |

**Figure 2A** Disease-free survival in all subtypes of HD according to LMP 1 expression

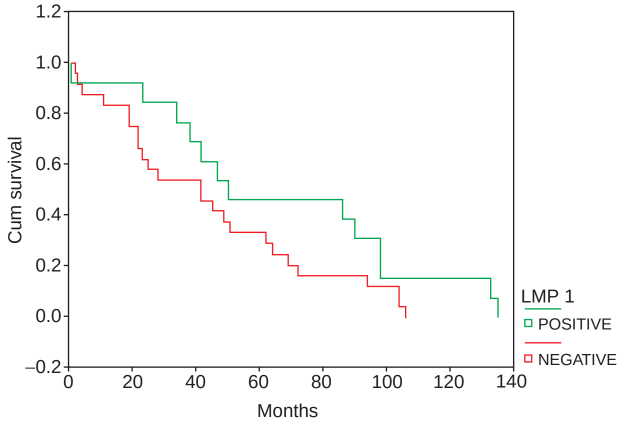

Log rank 0.54 $P = 0.46$

| LMP1 | mean | S.E. | 95% CI |
|---|---|---|---|
| negative | 51.6 ± 38 | 6 | 40 – 63 |
| positive | 63.1 ± 36.9 | 9 | 45 – 81 |

**Figure 2B** Disease-free survival in NS + MC subtypes according to LMP-1 expression

**Table 2** LMP1 expression according to stage and B symptoms

| | | B symptoms | | |
|---|---|---|---|---|
| **LMP1 expression** | **Stage** | **No** | **Yes** | **Total** |
| Negative | 1 + 2 | 21 | 8 | 29 |
| | 3 + 4 | 13 | 32 | 45 |
| Positive | 1 + 2 | 10 | 3 | 13 |
| | 3 + 4 | 8 | 5 | 11 |

longer disease-free survival ($P$ = 0.019) (Figure 4). Such significant differences were not observed in other subgroups divided by stage and B symptoms.

According to the multivariate regression analysis (Table 3), those characteristics which independently influenced the failure to achieve complete remission were: absence of LMP1 expression with odds ratio 6.4 (95%CI 0.024–0.89, $P$ = 0.04), subtype of HD with odds ratio 3.32 (95%CI 1.11–9.94 $P$ = 0.03) and older age in decade intervals with odds ratio 1.68 (95%CI 1.15–2.5 $P$ = 0.007) (Table 3). Cox's regression analysis model showed that older age (RR 2.75 95% CI 1.55–4.85 $P$ = 0.001) and HD subtype (RR 1.8 95% CI 1.06–2.98 $P$ = 0.03) also negatively influenced overall survival.

### DISCUSSION

This study presents the first analysis of EBV expression in HD from Croatia. We observed a bimodal age distribution as well as a predominance of the NS and MC subtypes (together comprising 85% of all cases). Such distributions are in accordance with data from developed countries (MacMahon, 1966; Glaser and Jarrett, 1996). Our results also confirm that LD HD is more common in older people and that this subtype is associated with adverse outcome, both in terms of lower complete remission rate and shorter disease free interval (Kaufman and Longo, 1992).

In the last few years, a number of studies from around the world have confirmed the presence of the EBV genome and expression of the EBV-encoded oncogene, LMP1, in the HRS cells of a proportion of HD patients. The frequency with which EBV is demonstrated in HD tumours shows geographical variability (Glaser and Jarrett, 1996; Glaser et al, 1997). Among Croatian adult patients with HD, we have demonstrated LMP1 expression in 26% of cases. This frequency is similar to that reported from other European countries (Pallesen et al, 1991; Herbst et al, 1992; Hummel et al, 1992; Murray et al, 1992; Glaser et al, 1997), including a study from Poland (Kordek et al, 1996). We have confirmed previous data showing that the NS subtype is often EBV negative (Jarrett et al, 1991; O'Grady et al, 1994). Although MC HD is considered to be an EBV-related disease, we did not find a significant association between LMP1 expression and MC disease. There have been few reports of the incidence of EBV in HD from Eastern Europe. In one Czech study MC rates of only 38% were recorded (Macak et al, 2000), although other studies from Poland (Kordek et al, 1996) have indicated higher EBV-positive rates for MC disease. Clearly, further larger samples are required to establish whether this and other studies reporting low EBV-positive rates for MC disease are outliers or are representative of true regional differences.

There are conflicting reports on the effect of EBV status on outcome in HD. Although two recent studies have shown no effect of EBV on outcome (Enblad et al, 1997; Axdorph et al, 1999) other studies have demonstrated that the presence of EBV in HRS cells is associated with a favourable outcome. Thus, two previous studies of adult HD from developed populations, have demonstrated improved overall survival (Morente et al, 1997) and higher complete response rates together with significantly longer disease-free interval (Murray et al, 1999) for patients with EBV-positive HD tumours. A more recent study of adult patients from India also showed that 10-year relapse-free survival was significantly higher for EBV-positive HD patients when compared to the EBV-negative group (Naresh et al, 2000). A second smaller study from South Africa of 36 children with HD showed that EBV-positive HD was associated with longer median survival times (Engel et al, 2000).

Our results showed that there were no statistically significant differences in stage, presence of B symptoms, as well as complete remission, disease-free survival and overall survival according to LMP1 expression either when all patients were examined or when

 *© 2001 Cancer Research Campaign*

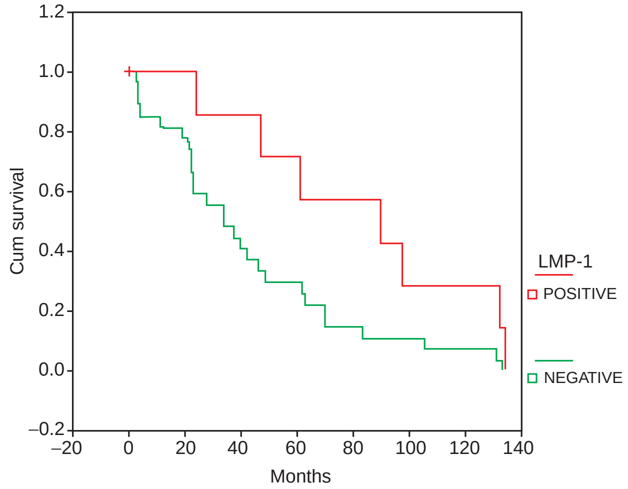

Log rank 5.21 $P = 0.02$

| LMP1 | mean | S.E. | 95% CI |
|---|---|---|---|
| negative | 44 ± 36.5 | 7 | 30–58 |
| positive | 94 ± 36 | 15 | 65–123 |

**Figure 3A** Disease-free survival in all subtypes of HD in age subgroup ≤ 30 years according to LMP 1 expression

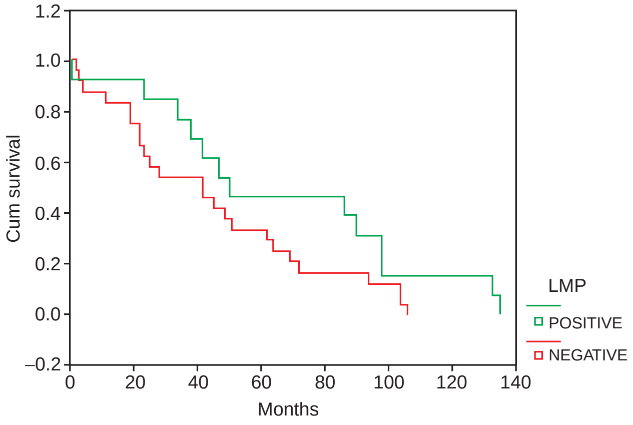

Log rank 3.69 $P = 0.05$

| LMP1 | mean | S.E. | 95% CI |
|---|---|---|---|
| negative | 48 ± 37.8 | 7 | 34–61 |
| positive | 85.4 ± 40 | 15 | 56–115 |

**Figure 3B** Disease-free survival in all subtypes of HD in age subgroup ≤ 34 years according to LMP 1 expression

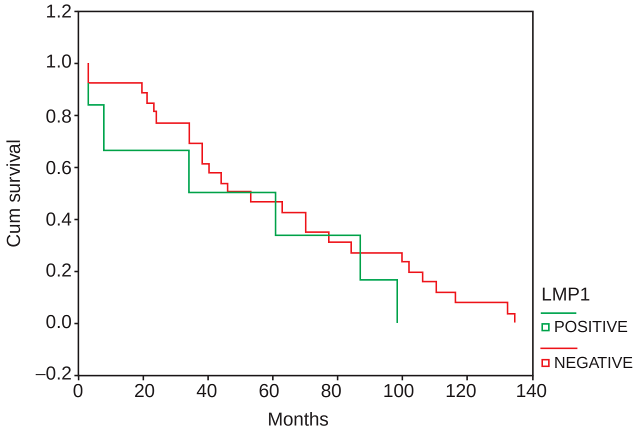

Log rank $P = 0.11$

| LMP1 | mean | S.E. | 95% CI |
|---|---|---|---|
| negative | 61.45 ± 34.8 | 8 | 46–77 |
| positive | 45.5 ± 35.7 | 10 | 26–65 |

**Figure 3C** Disease-free survival in all subtypes of HD in age subgroup > 35 years according to LMP 1 expression

LP and LD subtypes were excluded from the analysis. However, when the patients were subgrouped according to age, significantly longer disease-free survival was observed in LMP1-positive patients ≤ 30 years old as well as in patients ≤ 34 years old but not in older patients (> 35 years) where a non-significant trend towards longer disease-free survival of EBV-negative patients appeared. LMP1-negative patients aged >50 years also appeared to have longer disease-free survival indicating that reversal of the beneficial effect of EBV which seemed to occur after 35 years might also extend into the older age groups. This finding is supported by recent data from Glaser's group (Clarke et al, 2000) who assayed the EBV status of 311 women with Hodgkin's disease. In contrast to women in the 19–44 age group, older women (aged 45–79) with EBV-positive disease had significantly poorer survival than their EBV-negative counterparts. Clearly, a further study with sufficient numbers of cases in each age group is required to substantiate these trends. Furthermore, when the patients were subgrouped according to stage and presence of B symptoms, patients in early stage (1 + 2) disease without B symptoms had significantly better disease-free survival if they had LMP1-positive HRS cells. Overall, these data suggest that the clinical effects of EBV may be different at different ages or between different prognostic subgroups.

Despite these differences based on age and stage, EBV appeared to have an overall beneficial impact in our study population where failure to achieve complete remission was significantly associated with absence of LMP1 expression with odds ratio 6.4. At the present time we cannot precisely determine the factors that are

© 2001 Cancer Research Campaign

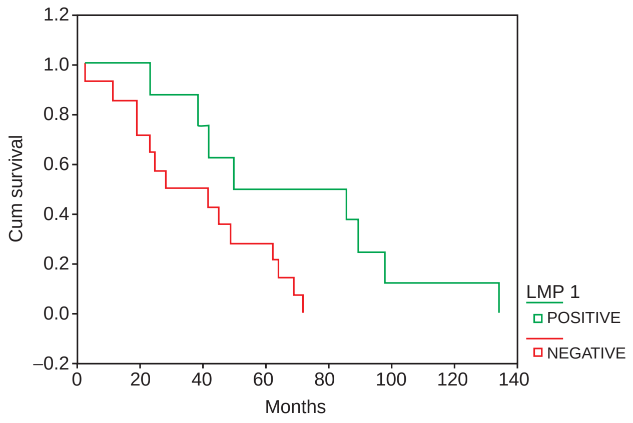

Log rank 5.49 *P* = 0.019

| LMP1 | mean | S.E. | Median | 95% CI |
|---|---|---|---|---|
| negative | 38 ± 23 | 6 | 28 | 26–50 |
| positive | 70 ± 38 | 13 | 50 | 44–97 |

**Figure 4** Disease-free survival in early stages (1 + 2) of HD without B symptoms according to LMP 1 expression responsible for the observed differences in prognosis between EBV-positive and -negative patients. Naresh et al (2000) have suggested that higher PCNA values in EBV-positive HRS cells may make them more responsive to chemotherapy- and radiotherapy-associated DNA damage. That HRS cells carrying the EBV genome might be more sensitive to chemotherapy-induced apoptosis is also supported by the observation that *bcl-2* is often overexpressed in EBV-negative, but not EBV-positive HRS cells (Jiwa et al, 1995) – a somewhat surprising finding when one considers the strong anti-apoptotic effects of LMP1 in vitro (Henderson et al, 1991; Wang et al, 1996).

LMP2 is also expressed by EBV-positive HRS cells (Niedobitek et al, 1997; Murray et al, 1998) and together with LMP1 is a target for cytotoxic T cells in association with different MHC class I restriction elements in vitro (Khanna et al, 1998; Lee et al, 1998). In vitro studies also show that HD cell lines can process and present epitopes from LMP1 and LMP2 in the context of multiple class I alleles and are sensitive to lysis by EBV-specific CTLs (Sing et al, 1997; Lee et al, 1998). Furthermore, EBV-specific CTLs can be generated from patients with HD, albeit at lower frequency than normal controls, and such cells survive and have antiviral activity in vivo (Roskrow et al, 1998). Thus, it is possible that the presence of EBV in HRS cells is able to elicit an immune response, which may in turn limit disease progression. These results are interesting since EBV-positive cases of HD have been shown to contain significantly more activated CTLs and express relatively higher levels of MHC class I than EBV-negative cases (Oudejans et al, 1996; Lee et al, 1998; Murray et al, 1998) Despite this, other studies have suggested that EBV-infected HRS cells may be unable to stimulate an effective anti-EBV CTL response. This is based on the observation that tumour-derived T lymphocytes from EBV-negative HD show EBV-specific cytotoxicity, whereas corresponding lymphocytes from EBV-positive HD lesions do not (Frisan et al, 1995), and is supported by the frequent overexpression of IL-10 in EBV-infected H-RS cells but not their EBV-negative counterparts (Herbst et al, 1996; Dukers et al, 2000).

It is possible that within the young adult group there is a beneficial EBV-specific immune response to the tumour cell population. In older patients this response may be less effective or other negative prognostic influences may override any beneficial effect EBV may have. The beneficial effects of EBV may also be more obvious in early stage disease and in 'A' patients where markers of poor prognosis are absent. The clinical effects of EBV in HD are therefore likely to be subtle and divergent in different subgroups of patients. This could well account for previous conflicting results, particularly when the majority of such studies have included small numbers or when the patients' treatment has been heterogeneous. Further studies are clearly required to establish the underlying biology responsible for the differing clinical effects of EBV in HD patients.

**Table 3** Factors influencing failure to achieve a complete remission

| Variable | Reference level | OR | 95% CI | *P* |
|---|---|---|---|---|
| LMP1 expression | | | | |
| Positive<br>Negative | LMP1 expression positive | 6.4 | 1.07–38.5 | 0.04 |
| Subtype of HD | | | | |
| NS<br>MC<br>LD | LP subtype | 3.32 | 1.11–9.94 | 0.03 |
| Age (years) | | | | |
| < 20<br>21–30<br>31–40<br>41–50<br>51–60<br>> 61 | < 20 years | 1.68 | 1.15–2.5 | 0.007 |

Model $\chi^2$ = 22.6, P = 0.01. OR = odds ratio. CI = confidence interval.

 *© 2001 Cancer Research Campaign*

### REFERENCES

Axdorph U, Porwit-MacDonald A, Sjoberg J, Grimfors G, Ekman M, Wang W, Biberfeld P and Bjorkholm M (1999) Epstein-Barr virus expression in Hodgkin's disease in relation to patient characteristics, serum factors and blood lymphocyte function. *Br J Cancer* **81**: 1182–1187

Clarke CA, Glaser SL, Dorfman RF, Mann RB, DiGiuseppe JA, Prehn A and Amninder RF (2000) Epstein-Barr virus and survival after Hodgkin's disease in a population-based series of women. *International Association for Research on Epstein–Barr virus and Associated diseases*. Ninth Biennial Conference. 22–27th June. Yale University, New Haven, USA

Claviez A, Tiemann M, Peters J, Kreipe H, Schneppenheim R and Parwaresch R (1994) The impact of EBV, proliferation rate, and Bcl-2 expression in Hodgkin's disease in childhood. *Ann Hematol* **68**: 61–66

Coates PJ, Mak WP, Slavin G and d'Ardenne AJ (1991) Detection of single copies of Epstein–Barr virus in paraffin wax sections by non-radioactive in situ hybridisation. *J Clin Pathol* **44**: 487–491

Cossman J, Messineo C and Bagg A (1998) Reed-Stemberg cell: survival in a hostile see. *Lab Invest* **78**: 229–235

Dukers DF, Jaspars LH, Vos W, Oudejans JJ, Hayes D, Cillessen S, Middeldorp JM and Meijer CJLM (2000) Quantitative immunohistochemical analysis of cytokine profiles in Epstein-Barr virus-positive and -negative cases of Hodgkin's disease. *J Pathol* **190**: 143–149

Enblad G, Sandvej K, Lennette E, Sundstrom C, Klein G, Glimelius B and Pallesen G (1997) Lack of correlation between EBV serology and presence of EBV in the Hodgkin and Reed-Sternberg cells of patients with Hodgkin's disease. *Int J Cancer* **72**: 394–397

Engel M, Essop MF, Close P, Hartley P, Pallesen G and Sinclair-Smith C (2000) Improved prognosis of Epstein-Barr virus associated childhood Hodgkin's lymphoma: study of 47 South African cases. *J clin Pathol* **53**: 182–186

Fellbaum C, Hansmann M-L, Niedermeyer H, Kraus I, Alavaikko MJ, Busch R, Putz B, Fischer R and Hofler H (1992) Influence of Epstein-Barr virus genomes on patient survival in Hodgkin's disease. *Am J Clin Pathol* **98**: 319–323

Frisan T, Sjoberg J, Dolcetti R, Boiocchi M, De Re V, Carbone A, Brautbar C, Battat S, Biberfeld P and Eckman M (1995) Local suppression of Epstein-Barr virus (EBV)-specific cytotoxicity in biopsies of EBV-positive Hodgkin's disease. *Blood* **86**: 1493–1501

Glaser SL and Jarrett RF The epidemiology of Hodgkin's disease (1996) *Baillieres Clin Haematol* **9**: 01–16

Glaser SL, Lin RJ, Stewart SL, Ambinder RF, Jarrett RF, Brousset P, Pallesen G, Gulley ML, Khan G, O'Grady J, Hummel M, Preciado MV, Knecht H, Chan JK and Claviez A (1997) Epstein-Barr virus-associated Hodgkin's disease: epidemiologic characteristics in international data. *Int J Cancer* **70**: 375–382

Glickman JN, Howe JG and Steitz JA. (1988) Structural analysis of EBER1 and EBER2 ribonucleoprotein particles present in Epstein-Barr virus infected cells. *J Virol* **62**: 902–911

Gruss H-J, Pinto A, Dystler J, Poppema S and Herrmann F (1997) Hodgkin's disease: a tumor with disturbed immunological pathways. *Immunol Today* **18**: 156–163

Gutensohn N and Cole P (1980) Epidemiology of Hodgkin's disease *Semin Oncol* **7**: 92–102

Henderson S, Rowe M, Gregory C, Croom-Carter D, Wang F, Longnecker R, Kieff E and Rickinson A (1991) Induction of bcl-2 expression by Epstein-Barr virus latent membrane protein 1 protects infected B cells from programmed cell death. *Cell* **65**: 1107–1115

Herbst H, Steinbecker E, Niedobitek G, Young LS, Brooks L, Muller-Lantzsch N and Stein H (1992). Distribution and phenotype of Epstein-barr virus-harboring cells in >Hodgkin's disease **80**: 484–91

Herbst H, Foss HD, Samol J, Araujo I, Klotzbach H, Krause H, Agathanggelou A, Niedobitek G and Stein H (1996) Frequent expression of interleukin-10 by Epstein-Barr virus-harboring tumor cells of Hodgkin's disease. *Blood* **87**: 2918–2929

Hummel M, Anagnostopoulos I, Dallenbach F, Korbjuhn P, Dimmler C and Stein H (1992) EBV infection patterns in Hodgkin's disease and normal lymphoid tissue: expression and cellular localization of gene products. *Brit J Haematol* **82**: 689–694

Hummel M, Ziemann K, Lammert H, Pileri S, Sabattini E and Stein H (1995) Hodgkin's disease with monoclonal and polyclonal populations of Reed-Sternberg cells. *N Engl J Med* **333**: 901–906

Jiwa NM, Oudejans JJ, Bai MC, van den Brule AJ, Horstman A, Vos A, van der Valk P, Kluin PM, Walboomers JM and Meijer CJ (1995) Expression of bcl-2 protein and transcription of the Epstein-Barr virus homologue BHRF-1 in Hodgkin's disease: implications for different pathogenic mechanisms. *Histopathology* **26**: 547–553

Kanzler H, Kuppers R, Hansmann ML and Rajewsky K (1996) Hodgkin and Reed-Sternberg cells in Hodgkin's disease represent the outgrowth of a dominant tumor clone derived from (crippled) germinal center B cells. *J Exp Med* **184**: 1495–1505

Kaufman D and Longo DL Hodgkin's disease (1992) *Crit Rev Oncol Hematol* **13**: 135–187

Khanna R, Burrows SR, Nicholls J and Poulsen LM (1998) Identification of cytotoxic T cell epitopes within Epstein-Barr virus (EBV) oncogene latent membrane protein 1 (LMP1): evidence for HLA A2 supertype-restricted immune recognition of EBV-infected cells by LMP1-specific cytotoxic T lymphocytes. *Eur J Immunol* **28**: 451–458

Kordek R, Jesionek-Kupnicka D, Biernat W and Wozniak L (1996) Expression of the latent membrane protein of Epstein-Barr virus in Hodgkin's disease. Age and subtype distribution in Polish patients. *Acta Haematol Pol* **27**: 15–20

Lee SP, Constandinou CM, Thomas WA, Croom-Carter D, Blake NW, Murray PG, Crocker J and Rickinson AB (1998) Antigen presenting phenotype of Hodgkin Reed-Sternberg cells: analysis of the HLA class I processing pathway and the effects of interleukin-10 on Epstein-Barr virus-specific cytotoxic T-cell recognition. *Blood* **92**: 1020–1030

Levine PH, Ablashi DV, Berard CW, Carbone PP, Waggoner DE and Malan L (1971) Elevated antibody titers to Epstein-Barr virus in Hodgkin's disease. *Cancer* **27**: 416–421

Macak J, Habanec B and Fabian P (2000) Detection of Epstein-Barr virus in Hodgkin's lymphoma. *Neoplasma* **47**: 156–161

MacMahon B Epidemiology of Hodgkin's disease (1966) *Cancer Res* **26**: 1189–1201

Morente MM, Piris MA, Abraira V, Acevedo A, Aguilera B, Bellas C, Fraga M, Garcia-Del-Moral R, Gomez-Marcos F, Menarguez J, Oliva H, Sanchez-Beato M and Montalban C (1997) Adverse clinical outcome in Hodgkin's disease is associated with loss of retinoblastoma protein expression, high Ki67 proliferation index, and absence of Epstein-Barr virus-latent membrane protein 1 expression. *Blood* **90**: 2429–2436

Mueller N, Evans A, Harris NL, Cornstock GW, Jellum E, Magnus K, Orentreich N, Polk BF and Vogelman J (1989) Hodgkin's disease and Epstein-Barr virus. Altered antibody pattern before diagnosis. *N Engl J Med* **32**: 689–695

Murray PG, Billingham LJ, Hassan HT, Flavell JR, Nelson PN, Scott K, Reynolds G, Constandinou CM, Kerr DJ, Devey EC, Crocker J and Young LS (1999) Effect of Epstein-Barr virus infection on response to chemotherapy and survival in Hodgkin's disease *Blood* **94**: 442–447

Murray PG, Constandinou CM, Crocker J, Young LS and Ambinder RF (1998) Analysis of major histocompatibility complex class I, TAP expression, and LMP2 epitope sequence in Epstein-Barr virus-positive Hodgkin's disease. *Blood* **92**: 2477–2483

Murray PG, Young LS, Rowe M and Crocker J (1992) Immunohistochemical demonstration of the Epstein-Barr virus encoded latent membrane protein in paraffin sections of Hodgkin's disease. *J Pathol* **166**: 1–5

Nakagomi H, Dolcetti R, Bejarano MT, Pisa P, Kiessling R and Masucci MG (1994) The Epstein-Barr virus latent membrane protein-1 (LMP1) induces interleukin-10 production in Burkitt lymphoma lines *Int J Cancer* **57**: 240–244

Naresh KN, Johnson J, Srinivas V, Soman CS, Saikia T, Advani SH, Badwe RA, Dinshaw KA, Muckaden M, Magrath I and Bhatia K (2000) Epstein-Barr virus association in classical Hodgkin's disease provides survival advantage to patients and correlates with higher expression of proliferation markers in Reed-Sternberg cells. *Ann Oncol* **11**: 91–96

Niedobitek G, Kremmer E, Herbst H, Whitehead L, Dawson CW, Niedobitek E, von Ostau C, Rooney N, Grässer FA and Young LS (1997) Immunohistochemical Detection of the Epstein-Barr Virus – Encoded Latent Membrane Protein 2A in Hodgkin's Disease and Infectious Mononucleosis. *Blood* **90**: 1664–1672

Oudejans JJ, Jiwa NM, Kummer JA, Horstman A, Vos W, Baak JP, Kluin PM, van der Valk P, Walboomers JM and Meijer CJLM (1996) Analysis of major histocompatibility complex class I expression on Reed-Sternberg cells in relation to the cytotoxic T-cell response in Epstein-Barr virus-positive and -negative Hodgkin's disease. *Blood* **87**: 3844–3851

Oudejans JJ, Jiwa NM and Meijer CJ (1997) Epstein-Barr virus in Hodgkin's disease: more than just an innocent bystander. *J Pathol* **181**: 353–356

Pallesen G, Hamilton-Dutoit SJ, Rowe M and Young LS (1991) Expression of Epstein-Barr virus latent gene products in tumour cells of Hodgkin's disease. *Lancet* **337**: 320–322

Roskrow MA, Suzuki N, Gan YJ, Sixbey JW, Ng CY, Kimbrough S, Hudson M, Brenner MK, Heslop HE and Rooney CM (1998) Epstein-Barr virus (EBV)-specific cytotoxic T lymphocytes for the treatment of patients with EBV-positive relapsed Hodgkin's disease. *Blood* **91**: 2925–2934

© 2001 Cancer Research Campaign 

Shibata D, Hansmann M-L, Weiss LM and Nathwani BN (1991) Epstein-Barr virus infections and Hodgkin's disease: a study of fixed tissue using the polymerase chain reaction. *Hum Pathol* **22**: 1262–1267

Sing A, Ambinder RF, Hokg DJ, Jensen M, Batten V, Petersdorf E and Greenberg PD (1997) Isolation of Epstein-Barr virus (EBV)-specific cytotoxic T-lymphocytes that lyse Reed-Sternberg cells: Implications for immune-mediated therapy for Hodgkin's disease. *Blood* **89**: 1978–1986

Vestlev PM, Pallesen G, Sandvej K, Hamilton-Dutoit SJ and Bendtzen SM (1992) Prognosis of Hodgkin's disease is not influenced by Epstein-Barr virus latent membrane protein. *Int J Cancer* **50**: 670–671

Wang D, Liebowitz D and Kieff E (1985) An EBV membrane protein expressed in immortalized lymphocytes transforms established rodent cells *Cell* **43**: 831–840

Wang S, Rowe M and Lundgren E (1996) Expression of the Epstein Barr virus transforming protein LMP1 causes a rapid and transient stimulation of the Bcl-2 homologue Mcl-1 levels in B-cell lines. *Cancer Res* **56**: 4610–4613

Weiss LM, Strickler JG, Warnke JA, Purtilo DT and Sklar J (1987) Epstein-Barr viral DNA in tissues of Hodgkin's disease. *Am J Pathol* **129**: 86–91

Wu TC, Mann RB, Charache P, Hayward SD, Staal S, Lambe BC and Ambinder RF (1990) Detection of EBV gene expression in Reed-Sternberg cells of Hodgkin's disease. *Int J Cancer* **46**: 801–804

*© 2001 Cancer Research Campaign*
